# Influencing the Influencers: How Health Experts Are Partnering With Content Creators to Fight Misinformation Online

**DOI:** 10.2196/93450

**Published:** 2026-02-27

**Authors:** Wendy Glauser

**Keywords:** social media, opinion leaders, health communication, information dissemination, misinformation, public health, health promotion

**Key Takeaways**

- By offering training and support to content creators, public health advocates can improve the quality of health information on social media platforms.
- Partnering with content creators with diverse audiences can help health communicators reach populations they’re struggling to engage.

*Scroll*. “Five essential questions that you must ask your surgeon prior to any operation.”

*Click*. “Yesterday, I posted a picture of when I started on Ozempic and four weeks after Ozempic and many of you said....”

*Swipe*. “The World Health Organization lists processed meats like bacon, ham, salami and sausages in the same category as cigarettes and asbestos. But what does that even mean?”

These are three real examples [1-3] of the millions or potentially billions of TikTok videos that contain health information. In a Kaiser Family Foundation survey of over 1000 adults who were selected to be representative of the US population, 55% said they use social media to find health information at least occasionally [4].

Social media creators can influence what people eat, how they exercise, what medications they take or don’t take, whether they seek out immunizations and preventive health screenings, and the list goes on. Whether that influence helps or harms depends in part on the accuracy of the information shared, and that information is not always accurate. For example, in a study that analyzed 1000 TikTok videos on mental health, 6.3% contained disinformation, which was defined as providing false or scientifically unsupported information, and 15.7% contained misinformation, which was defined as providing only partially supported information [5].

Realizing the potential impact, public health and health communication experts have begun to partner with content creators to fight misinformation online. As Matthew Motta, PhD—associate professor of Health Law, Policy and Management at the Boston University School of Public Health—explains, “If you can partner with just a handful of social media content creators, you might be able to reach millions of people with your message.”

## Collaboration Between Health Experts and Influencers

Three years ago, Amanda Yarnell, MS—senior director of the Harvard T.H. Chan School of Public Health’s Center for Health Communication—launched the Creator Program [6] to help social media creators “counter health misinformation and spread evidence-based science to their communities,” as the program’s website describes. Yarnell explains that, at the time, “there was very little information, even about the health information creators shared.” In the past few years, more research evaluating patterns in the content, style, and tone of health information shared on social media has emerged, but there are still very few health researchers attempting to intervene in those conversations by engaging creators.

While other programs have popped up in recent years that support health providers and scientists who are also creators, such as the World Health Organization’s Fides program [7] or the Evidence Collective [8], the Creator Program is unique in that it also collaborates with influencers who don’t necessarily have health or science degrees.

The impact of the Creator Program’s initiatives is being studied. In one 2024 study [9], 105 English-language creators, who collectively reached about 17 million followers, were randomly assigned to a control group, which received asynchronous digital tool kits summarizing evidence-based mental health information, or a group that received the tool kits plus optional virtual training sessions. Researchers analyzed 3465 videos posted before and after the intervention. Post intervention, 26% of the creators who received tool kits and training invites, versus 21% of the control group, referenced the evidence-based themes included in the toolkit. The authors wrote that “although these effects are relatively small in substantive size, their reach,” given the size of their audiences,“may be comparatively larger.”

In another study (currently a preprint), Motta and colleagues [10] engaged social media influencer Kenzie Brenna to create two videos on how to respond when a peer is experiencing emotional distress; one was created before a virtual training program delivered by mental health experts, and one was created afterward. A demographically representative sample of 1000 US youths between the ages of 14 and 22 years were then randomly assigned to view either video. The effect was modest but significant: 80% of those who viewed the posttraining video described it as informative (vs 60% of those who viewed the pretraining video), and more people in the group who viewed the posttraining video said that they felt confident in providing emotional support to a distressed peer.

Sasha Hamdani, MD—a content creator and psychiatrist—took part in the Creator Program virtual training sessions and also received a toolkit. Being part of the Creator Program, she says, “reinforced that even the most accurate data can be misunderstood if it is not communicated clearly and responsibly.” Hamdani, who has about a million followers on TikTok and over 930,000 followers on Instagram, shares videos on mental health, especially attention-deficit/hyperactivity disorder (ADHD). She says being part of the Creator Program reminded her that “good health communication reduces misinformation by being precise, transparent about limitations, and intentional about language, especially in digital spaces where content travels quickly.”

## Reaching More People, More Often

Engaging with social media influencers allows public health experts to reach more people and “target diverse folks that public health organizations have failed at reaching, such as young people, as well as Black, Brown, Indigenous, and LGBTQ people,” says Yarnell. Many of the influencers in the Creator Program, for example, have audiences that include a large proportion of people from one or more of these groups.

When it comes to prebunking (preparing people to resist misleading claims before they’re exposed to them) and debunking (countering falsehoods after exposure), social media is the tool that “has the most promise currently,” says Shaon Lahiri, PhD—assistant professor of public health at the College of Charleston in South Carolina. “It’s where the misinformation is coming from,” he explains. Plus, by virtue of engaging with audiences daily, creators can share similar information multiple times in different ways, or as Lahiri describes it, they can create “booster shots” to better inoculate people against misinformation.

## More Engagement, Less Misinformation

Popular content creators tend to use methods that are engaging and impactful, as they rely on their metrics to learn what resonates. People are more persuaded by messaging that is “fun and not very heavy-handed,” Lahiri explains. The technology capabilities in the social media platforms themselves also lend well to misinformation debunking/prebunking. For example, “you can do a Stitch on TikTok where you briefly show the misinformation and then you explain what’s wrong with it,” Lahiri explains, referring to taking a short clip from someone else’s post and adding it to the beginning of your own.

Motta recognizes that these programs compete with billion-dollar companies and “social media companies that have really dropped the ball” in reducing misinformation. Still, he says, “there are plenty of content creators who want to fight against the spread of misinformation on social media platforms. These same creators are also really good at figuring out how to make sure that information permeates through.”

Motta adds that even though social media videos are too often inaccurate, the vast majority of creators aren’t intentionally sharing misleading information. As misinformation continues to travel faster than evidence, engaging the people who already command attention online may be the best way for public health to keep up.
